# A Systematic Review and Meta-analysis of Optimized CMV Preemptive Therapy and Antiviral Prophylaxis for CMV Disease Prevention in CMV High-Risk (D+R-) Kidney Transplant Recipients

**DOI:** 10.1097/TXD.0000000000001514

**Published:** 2023-07-12

**Authors:** Lakshin Kumar, Cristina Murray-Krezan, Nina Singh, Daniel C. Brennan, Robert M. Rakita, Sayan Dasgupta, Cynthia E. Fisher, Ajit P. Limaye

**Affiliations:** 1 Division of Allergy and Infectious Diseases, Department of Medicine, University of Washington, Seattle, WA.; 2 Division of General Internal Medicine, Department of Medicine, University of Pittsburgh School of Medicine, Pittsburgh, PA.; 3 Department of Medicine, VA Pittsburgh Healthcare System and University of Pittsburgh, Pittsburgh, PA.; 4 Division of Nephrology, Department of Medicine, Johns Hopkins University School of Medicine, Baltimore, MD.; 5 Vaccine and Infectious Disease Division, Fred Hutchinson Cancer Center, Seattle, WA.

## Abstract

The optimal strategy for cytomegalovirus (CMV) disease prevention in CMV donor/recipient kidney transplant recipients remains uncertain. Conclusions of prior meta-analyses that CMV disease rates with preemptive therapy (PET) and universal prophylaxis (UP) were comparable may have been affected by inclusion of studies lacking key determinants of efficacy of the respective strategies.

**Methods.:**

We conducted a systematic review and meta-analysis of PET with weekly CMV polymerase chain reaction monitoring for ≥3 mo and UP with 6 mo of valganciclovir. PubMed and Embase databases were reviewed from January 1, 2010, to April 1, 2022. Risk of bias was assessed with 3 instruments (Cochrane RoB, Cochrane RoBINS-I, and an instrument for assessing risk in observational studies). The primary outcome was CMV disease incidence by 1-y posttransplant. Secondary outcomes by 1-y were graft loss, acute allograft rejection, and mortality. Results were synthesized using generalized linear mixed model meta-analysis. PET studies were stratified into low-threshold (LT) and high-threshold (HT) PET based on the viral load threshold for initiation of antiviral therapy.

**Results.:**

Twenty-five studies met inclusion criteria (6 PET, 19 UP). CMV disease incidence was significantly higher in HT (0.30 [95% confidence interval (CI), 0.22-0.39]) versus LT PET (0.06 [95% CI, 0.03-0.12]). LT PET was associated with a significantly lower CMV disease incidence (0.06 [95% CI, 0.03-0.12]) versus UP (0.21 [95% CI, 0.17-0.27]). Incidence of graft loss, acute allograft rejection, or mortality was not significantly different between LT PET and UP (*P* > 0.05 for all comparisons). Receipt of lymphocyte-depleting antibodies was not associated with a significant difference in CMV disease incidence (odds ratio = 1.34 [95% CI, 0.80-2.25]).

**Conclusions.:**

LT PET is associated with a significantly lower incidence of CMV disease compared to UP with similar rates of other clinical outcomes. These findings provide rationale and preliminary data for a randomized superiority trial of optimized LT-PET versus UP in donor seropositive recipient seronegative kidney transplant recipients.

Cytomegalovirus (CMV) disease remains an important cause of morbidity, mortality, and increased costs in kidney transplant recipients (KTRs) despite current preventive strategies and disproportionately affects high-risk CMV serostatus (donor seropositive recipient seronegative [D+R-]) patients.^1^ The potential advantages and disadvantages of the 2 CMV disease prevention strategies, preemptive therapy (PET) and universal prophylaxis (UP), have been reviewed in detail elsewhere.^2-4^ Current guidelines generally favor UP over PET, especially in high-risk CMV D+R- KTRs and those receiving lymphocyte-depleting immunosuppression, in part because of logistical concerns with PET (need for frequent monitoring, need for prompt initiation of antiviral therapy [AVT], and uncertainty about the viral threshold for initiating AVT).^2,4,5^

There are few direct comparisons of PET and UP in CMV D+R- KTRs. Meta-analyses of efficacy between the 2 CMV disease prevention strategies have generally concluded that PET and UP were comparably effective for preventing CMV disease.^3,6-12^ However, major improvements in PET and UP strategies have occurred since these studies were published, including the use of valganciclovir (VGCV) over oral ganciclovir (GCV) and 200-d rather than 100-d duration of prophylaxis.^13^ For PET, the use of more sensitive quantitative assays for CMV detection (polymerase chain reaction [PCR] for the presence of DNA in blood [DNAemia] rather than pp65 antigenemia), weekly monitoring for at least 100-d, use of a low viral load threshold for initiation of AVT, and use of full treatment doses of either VGCV or intravenous GCV have all been proposed to improve the efficacy of the strategy.^2,14,15^ Inclusion of PET and UP studies without these key determinants of efficacy may have affected results in prior meta-analyses.

A recent direct head-to-head randomized controlled clinical trial of PET and UP in high-risk CMV D+R- liver transplant recipients demonstrated superiority of PET for prevention of CMV disease with comparable rates of other clinical outcomes (bacterial and fungal infections, acute allograft rejection, and graft and patient survival).^16^ The study used an optimized PET protocol consisting of a sensitive CMV DNAemia assay, weekly monitoring for 100-d posttransplant, and a low viral load threshold for initiation of PET (AVT initiated at any level of detected CMV DNAemia). PET led to significantly enhanced CMV-specific immune responses compared with UP, providing a potential mechanistic basis for the reduction in late-onset CMV disease with PET.

Although there are important differences in immunosuppression and other clinical aspects between liver and kidney transplants, based on these results and for the reasons described above, we performed a systematic review and meta-analysis of PET and UP for CMV disease prevention in CMV D+R- KTRs, restricted to studies that incorporated key determinants of efficacy for each of the respective CMV disease prevention strategies.

## MATERIALS AND METHODS

### Literature Search

We systematically queried the Embase and PubMed databases for studies of adult KTRs that included CMV D+R- patients conducted between January 1, 2010, and April 1, 2022. This timeframe was chosen to represent contemporary immunosuppression regimens and relatively consistent practices for CMV diagnostics and therapeutic strategies. The details of the search strategy are shown in Figure S1 (SDC, http://links.lww.com/TXD/A551). Study inclusion criteria were:

For all studies:

1. At least 5 CMV D+R- serostatus KTRs.
2. Primarily adult population.
3. CMV disease recorded at 1-y posttransplant.

For studies of PET:

1. Patients were monitored for CMV DNAemia using a quantitative PCR (qPCR) assay (those using pp65 antigenemia were excluded because of previously shown inferior sensitivity of pp65 compared with PCR).^17,18^
2. CMV monitoring was conducted at least weekly for a minimum duration of 3 mo or 100 d posttransplant.
3. Upon detection of CMV DNAemia at or above the protocol-specified threshold level, treatment doses of VGCV (900 mg b.i.d) or intravenous GCV (5 mg/kg b.i.d), or renally adjusted equivalent, were administered.

For studies of UP:

1. Antiviral prophylaxis duration of 6 mo or 180-200 d (studies using 100-d prophylaxis were excluded because of previous results indicating inferiority of 100-d prophylaxis).^13^
2. Antiviral prophylaxis drug was VGCV or intravenous GCV (most common and recommended).^2^
3. Standard Food and Drug Administration label-specified prophylactic dosing of antiviral drug (900 mg q.d. for VGCV or 5 mg/kg q.d. intravenously for GCV, or renal function-adjusted equivalent).

Initially, 2 authors (L.K. and A.P.L.) independently reviewed the titles and abstracts of all studies. If the study was unable to be excluded based on the abstract or title, and either PET or prophylaxis was mentioned for CMV prevention in the abstract, the full text was independently reviewed in detail for potential inclusion. In the case of disagreement over inclusion of a study, consensus was reached through discussion. Duplicates were removed using SR Accelerator’s Deduplicator (Bond University). For studies that qualified for full-text review but were excluded, the first identified exclusion criterion was recorded.

Data extraction was conducted using a prespecified and standardized set of outcomes. Authors of included studies that did not report the outcomes of interest were contacted to obtain missing data.

Because PET using a low threshold for AVT initiation (defined as any level of detectable CMV DNAemia) was shown to be superior to UP for CMV disease prevention in CMV D+R- liver transplant recipients, we further subcategorized PET studies into low-threshold (LT) and high-threshold (HT) PET subgroups. LT PET was defined as initiation of AVT at any level of CMV DNAemia, whereas HT PET was defined as initiation of AVT at a prespecified level of CMV DNAemia that was higher than the lower limit of detection of the qPCR assay used in the study. Details of the PET protocol including the assay used and threshold for AVT initiation are shown in Table S1 (SDC, http://links.lww.com/TXD/A551). Three PET studies used whole blood and three used plasma samples for CMV PCR monitoring.^19-21^ To estimate comparable CMV loads between whole blood and plasma qPCR studies, we used regression from previously published studies that directly compared concomitant plasma and whole blood CMV DNAemia levels.^22,23^ As shown in Table S1 (SDC, http://links.lww.com/TXD/A551), the estimated threshold for AVT initiation in the HT PET studies was approximately 1 log-fold higher than the threshold used for AVT initiation in LT PET studies.

This systematic review was done in accordance with the Preferred Reporting Items for Systematic Reviews and Meta-Analyses guidelines (Figure S2, SDC, http://links.lww.com/TXD/A551) but was not registered in The International Prospective Register of Systematic Reviews, because a protocol was not prepared for publication before analysis. Data regarding the systematic review process and evidence used in the analyses are available upon request.

### Outcomes

The primary outcome was incidence of CMV disease (either CMV syndrome or end-organ disease). Secondary outcomes were the incidence of transmitted CMV infection among PET studies (as measured by incidence of CMV DNAemia at any level during the monitoring period), allograft survival, acute allograft rejection, and patient survival. An exploratory outcome was the effect of induction immunosuppression with lymphocyte-depleting therapy on the outcome of CMV disease. We limited the analysis to outcomes by 1-y posttransplant to ensure consistent follow-up across studies.

### Risk of Bias Assessment

Risk of bias assessment included study characteristics, study design (prospective cohort study, retrospective cohort study, case-control study, or randomized controlled trial), and cohort size, and was assessed using 3 different instruments. For randomized controlled trials, we used the Cochrane “Risk of Bias 2” tool,^24^ for nonrandomized trials of interventions, we used “Risk of Bias in Nonrandomized Studies of Interventions” tool,^25^ and for observational studies in which there were no interventions, we used an instrument reported in Munn et al^26^ created specifically for studies of prevalence. Risk of bias was not assessed in 1 study because it was a post hoc analysis of a study for which the design was unavailable.^27^

### Statistical Methodology

Data were grouped according to CMV prevention strategy (LT PET, HT PET, or UP), and meta-analysis was conducted for each outcome. Data were visualized using forest plots.

For binary outcomes without a comparator (CMV disease incidence, incidence of secondary clinical outcomes), proportional meta-analysis was conducted using the incidence of the outcome. For binary outcomes with a comparator (CMV disease risk with and without lymphocyte-depleting antibody induction [LDA]), an odds ratio (OR) was calculated for each study and used to conduct meta-analysis. Previous studies have identified limitations in interpretation and coverage using both the logit transformation and the popular Freeman-Tukey double arcsin transformation for analysis of single proportions.^28-30^ Thus, all meta-analyses were conducted using a generalized linear mixed model as described in Lin et al.^31^ We estimated 95% confidence intervals (CIs) using the Wilson method.^32^ For zero event outcomes, 95% CIs were estimated using Hanley’s Rule of Three.^33^ Heterogeneity was quantified using the *I*^2^ value, which describes the percentage of variability across the included studies not attributable to sampling error, and τ^2^ which denotes the estimated variance in true effect sizes between studies. The significance of differences in outcomes between the UP, HT-PET, and LT-PET subgroups was tested using the Cochran Q statistic, which assesses the effect of heterogeneity between the included studies and is compared with a Chi-square distribution with N—1 degrees of freedom, where N represents the number of studies. If significant differences were found, a meta-regression was performed with the UP subgroup as the baseline.

Publication bias in the primary outcome was assessed in the UP and LT PET cohorts using the Doi Plot and the LFK index.^34^ There were insufficient studies to separately assess publication bias in the HT PET subgroup. The Doi Plot is an analog of the funnel plot that more accurately depicts visual asymmetry and publication bias. In the Doi Plot, a study’s normalized quantile, represented by the absolute value of the quantile’s *z* score, is plotted against an effect size, in this case, CMV disease incidence. A lack of publication bias would indicate that studies representing quantiles both above and below the middle quantile would deviate from the median effect size with similar magnitude, leading to a highly symmetrical plot, whereas the presence of publication bias would introduce asymmetry. The degree of asymmetry can be quantified using the LFK index, which represents the numerical difference between the area under the 2 sides of the Doi Curve.

All analyses, including data cleaning, meta-analysis, and figure creation, were conducted in R (v 4.1.2) using the packages Tidyverse (v 1.3.1), and meta (v 6.2.1).

## RESULTS

### Study Selection

**FIGURE 1.**
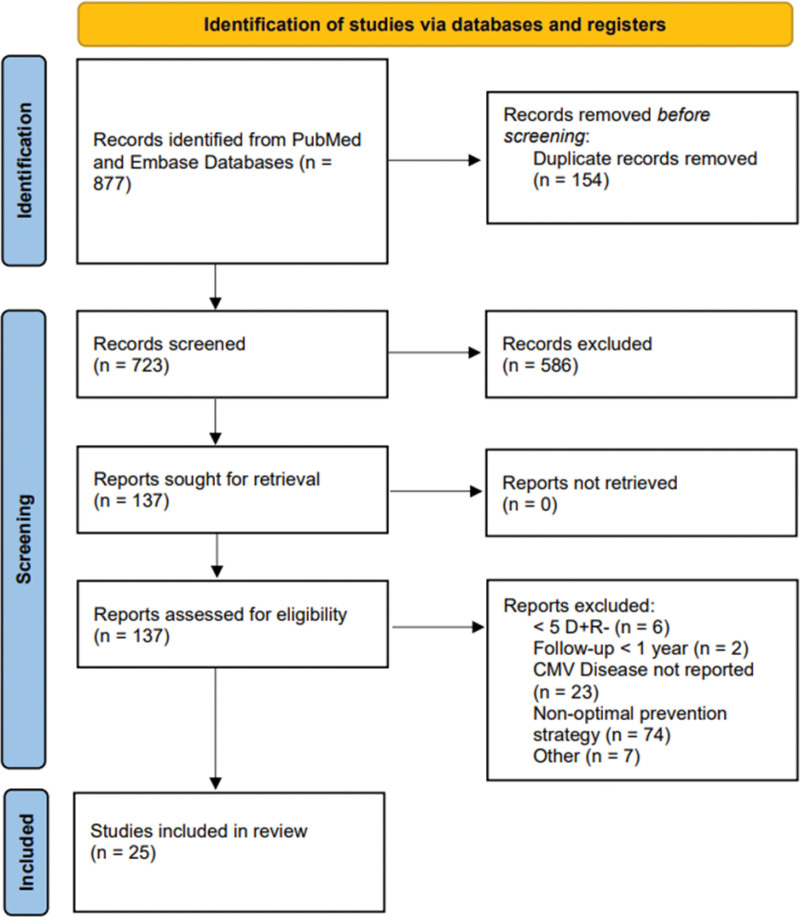
Flowchart of the data extraction process. CMV, cytomegalovirus; D+R-, donor seropositive recipient seronegative.

Among 877 studies screened, 154 duplicates and 586 studies were excluded from the title/abstract, leaving 137 studies that included 149 cohorts (115 UP and 34 PET) that were fully reviewed for eligibility by 2 independent authors (Figure 1). Among these 137 studies, 19 UP studies^13,27,35-51^ and 6 PET studies^19-21,52-54^ met inclusion criteria, comprising 993 patients in the UP cohort and 234 patients in the PET cohort. One study that met inclusion criteria was excluded because of variable definitions of CMV disease and overlap of cohorts from the same center in 2 separate publications.^55^ Characteristics of included studies are shown in Table 1. Among the 6 PET studies, 4 were subcategorized as LT-PET (n = 114) and 2 as HT-PET (n = 120).

**TABLE 1.** Characteristics of included studies.

Study	Location	No. D+R- patients	CMV disease, n	Graft loss, n	Death, n	Acute rejection, n	CMV prevention strategy	CMV disease definition(s) used
UP studies								
Humar et al (2010)	Multicenter	155	25	3	0	17	UP	Humar et al (2006)
Leone et al (2010)	France	67	15	NR	NR	NR	UP	Humar et al (2006)
Abate et al (2010)	Italy	13	1	0	0	1	UP	No definition listed
Boudreault et al (2011)	USA	34	11	0	1	12	UP	Humar et al (2006)
Abate et al (2013)	Italy	20	1	NR	0	NR	UP	Custom definition
Manuel et al (2013)	Switzerland	28	7	3	1	NR	UP	Humar et al (2006)
Posadas Salas et al (2013)	USA	44	2	4	1	0	UP	Custom definition
Gabardi et al (2015)	USA	107	26	3	3	14	UP	Custom definition
Stevens et al (2015)	USA	45	5	1	1	4	UP	Humar et al (2006)
Perez-Jacoiste Asin et al (2016)	Spain	8	4	0	0	0	UP	Humar et al (2006)
Puttarajappa et al (2016)	USA	42	14	2	2	17	UP	No definition listed
Fleming et al (2017)	USA	17	3	NR	NR	NR	UP	Custom definition
Freedman et al (2019)	USA	138	35	4	4	16	UP	Ramanan et al (2013) and Kotton et al (2018)
Perez-Flores et al (2019)	Spain	23	8	0	0	6	UP	Ljungman et al (2017)
Andreani et al (2020)	France	12	4	NR	NR	0	UP	Humar et al (2006)
Hellemans et al (2021)	Belgium	40	5	0	0	2	UP	Razonable and Blumberg (2015)
Nowak et al (2021)	Germany	56	10	NR	1	18	UP	Ljungman et al (2017)
Raiha et al (2021)	Finland	136/481^*a*^	52	14	9	56	UP	Razonable et al (2019) and Ljungman et al (2017)
Aboujaoude et al (2021)	Lebanon	8	2	0	0	NR	UP	No definition listed
PET studies								
HT PET studies								
Couzi et al (2012)	France	80	21	8	0	16	HT PET	Humar et al (2006)
Atabani et al (2012)	UK	40	15	NR	NR	4	HT PET	Ljungman et al (2002) and Kotton et al (2010)
LT PET studies								
van der Beek et al (2010)	The Netherlands	35	1	1	0	NR	LT PET	Ljungman et al (2002)
Martin-Gandul et al (2014)	Spain	23	2	0	0	3	LT PET	Ljungman et al (2002); de La Torre Cisneros et al (2011); Kotton et al (2013)
Cantisan et al (2016)	Spain	25	3	NR	0	NR	LT PET	de la Torre-Cisneros et al (2011)
Lumley et al (2019)	UK	31	1	0	0	NR	LT PET	Ljungman et al (2017)

^*a*^Raiha et al (2021) examined two cohorts: a cohort of 481 D+R- KTRs in which clinical outcomes (graft loss, acute rejection, and mortality) were studied, and a subcohort of 136 patients in which CMV disease incidence was collected.

CMV, cytomegalovirus; D+R-, donor seropositive recipient seronegative; HT, high threshold; KTR, kidney transplant recipient; LT, low threshold; NR, not reported; PET, preemptive therapy; UP, universal prophylaxis.

### Risk of Bias in Selected Studies

Risk of bias for the outcome of CMV disease are shown in Tables S2–S4 (SDC, http://links.lww.com/TXD/A551–S4, SDC, http://links.lww.com/TXD/A551), classified into randomized-controlled trials (Table S2, SDC, http://links.lww.com/TXD/A551), nonrandomized studies of interventions (Table S3, SDC, http://links.lww.com/TXD/A551), and observational studies (Table S4, SDC, http://links.lww.com/TXD/A551). Most nonrandomized interventional trials carried a moderate risk of bias as a result of confounding and measurement of outcomes.^25^ Four studies (all UP) exhibited a high/serious risk of bias. Of these 4 studies, 2 were because of a very low sample size,^42,51^ whereas the other 2 were because of methodological concerns in which patients were not enrolled immediately after transplant.^36,38^

### Primary Outcome: CMV Disease by 1-y Posttransplant

**FIGURE 2.**
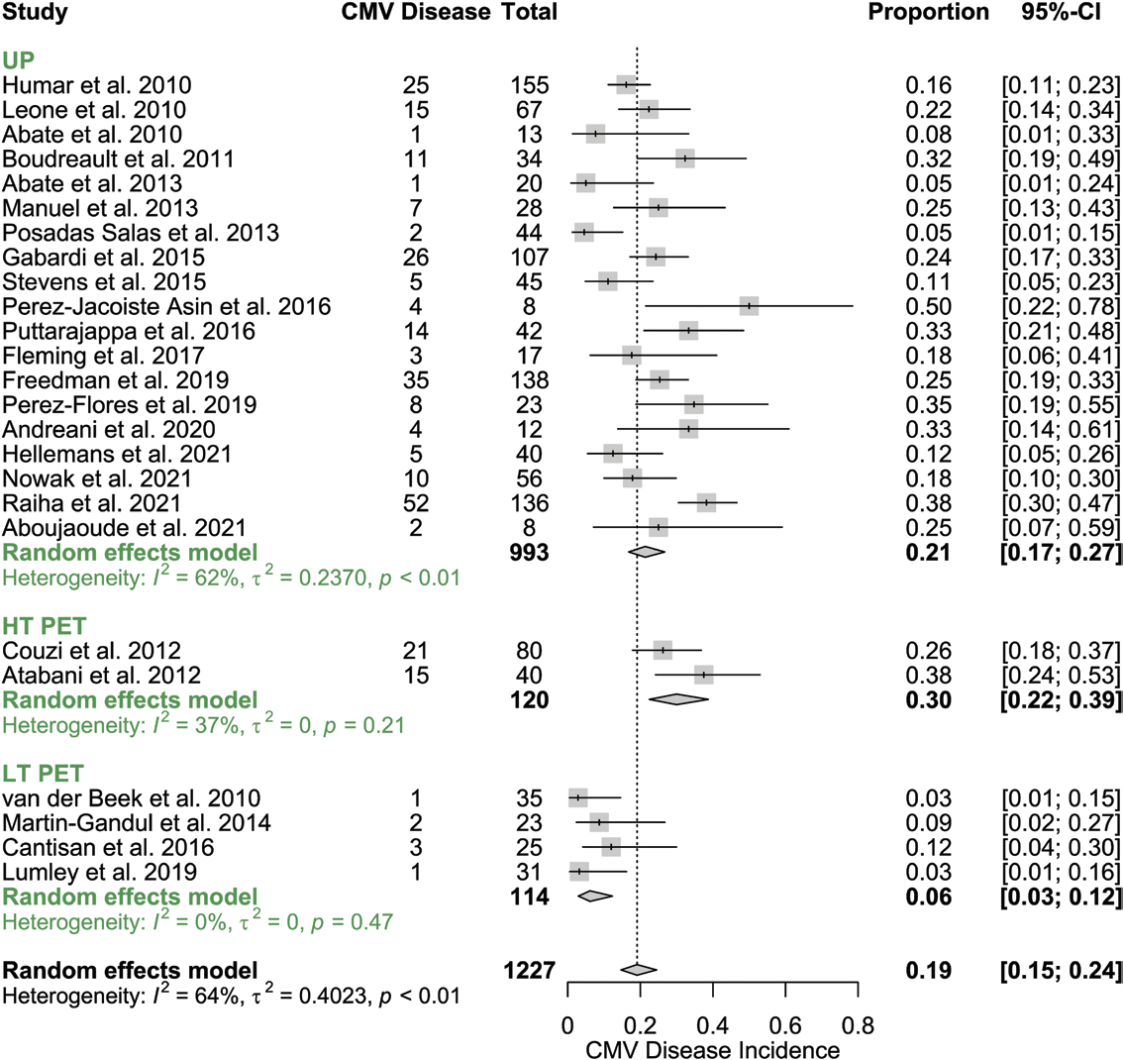
Forest plot showing meta-analysis of primary outcome: CMV disease incidence at 12-mo posttransplant in all studies by CMV prevention strategy. CI, confidence interval; CMV, cytomegalovirus; HT, high threshold; LT, low threshold; PET, preemptive therapy; UP, universal prophylaxis.

The incidence of CMV disease by 1-y posttransplant, according to CMV disease prevention strategy, is shown in Figure 2. Heterogeneity was classified as low within the LT PET and HT PET subgroups, and CMV disease incidence was significantly higher in HT PET (0.30 [95% CI, 0.22-0.39]) compared with LT PET (0.06 [95% CI, 0.03-0.12]; *P* < 0.001).

UP studies showed moderate-high heterogeneity (I^2^ = 61.8% [95% CI, 37.1%-76.8%]; Q = 56.72; *P* < 0.0001). There were significant differences in CMV disease incidence among the 3 subgroups (HT PET, LT PET, and UP) (Q = 18.5, *P* < 0.0001). Receipt of LT PET was associated with a significantly lower CMV disease incidence compared with the referent UP group $\left(\hat{\beta }=-1.483,\;P=0.002\right)$. HT PET was not significantly associated with a higher CMV disease incidence compared with UP $\left(\hat{\beta }=0.4884,\;P=0.21\right)$.

### Effect of Lymphocyte-depleting Induction Immunosuppression Therapy on Incidence of CMV Disease

**FIGURE 3.**
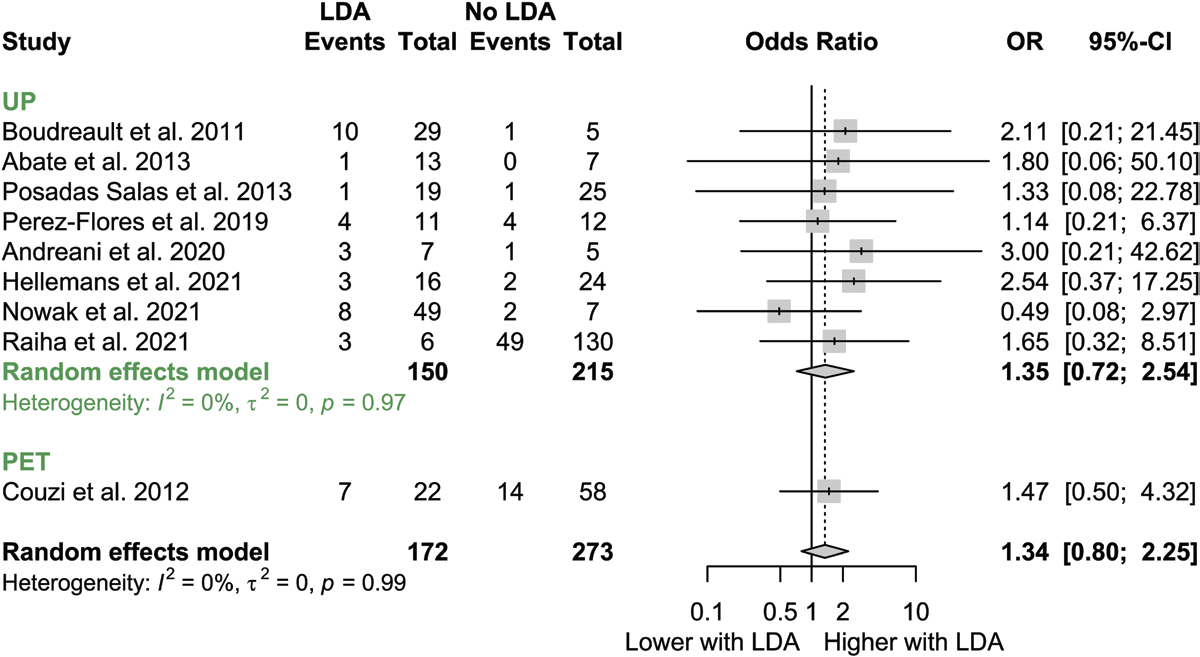
Forest plot of ORs of developing CMV disease stratified by receipt of lymphocyte-depleting induction therapy. CI, confidence interval; CMV, cytomegalovirus; LDA, lymphocyte-depleting antibody; OR, odds ratio; PET, preemptive therapy; UP, universal prophylaxis.

CMV disease incidence stratified by receipt of LDA was available for 12 of 19 UP cohorts, 3 of 4 LT-PET cohorts, and 2 of 2 HT-PET cohorts. Among these 17 studies, 8 studies in which either no patients or all patients received LDA were excluded. As shown in Figure 3 the overall OR of developing CMV disease was not significantly different among those who received LDA and those who did not (OR = 1.34 [95% CI, 0.80-2.25]; *z* = 1.1; *P* = 0.266).

### Donor to Recipient CMV Transmission With PET as Assessed by Detection of CMV DNAemia at any Level During the Monitoring Period

The pooled incidence of CMV transmission was 0.65 (129 of 199 [95% CI, 0.58-0.71]) and similar between the HT and LT subgroups of PET studies (Table 2). The incidence of CMV transmission could not be determined among UP studies because routine monitoring for CMV DNAemia was not consistently performed during prophylaxis.

**TABLE 2.** Proportion of patients who developed DNAemia in selected PET studies.

Study	DNAemia incidence (no. cases/no. participants)
HT-PET	
Couzi et al (2012)	0.60 (48/80)
Atabani et al (2012)	0.70 (28/40)
HT-PET total	0.63 (76/120)
LT-PET	
Martin-Gandul et al (2014)	0.87 (20/23)
Cantisan et al (2016)	0.64 (16/25)
Lumley et al (2019)	0.54 (17/31)
LT-PET total	0.67 (53/79)
Total PET	0.65 (129/199), 95% CI, 0.58-0.71

CI, confidence interval; DNAemia, presence of DNA in blood; HT, high threshold; LT, low threshold; PET, preemptive therapy.

### Secondary Outcomes: Mortality, Graft Loss, and Acute Allograft Rejection

**FIGURE 4.**
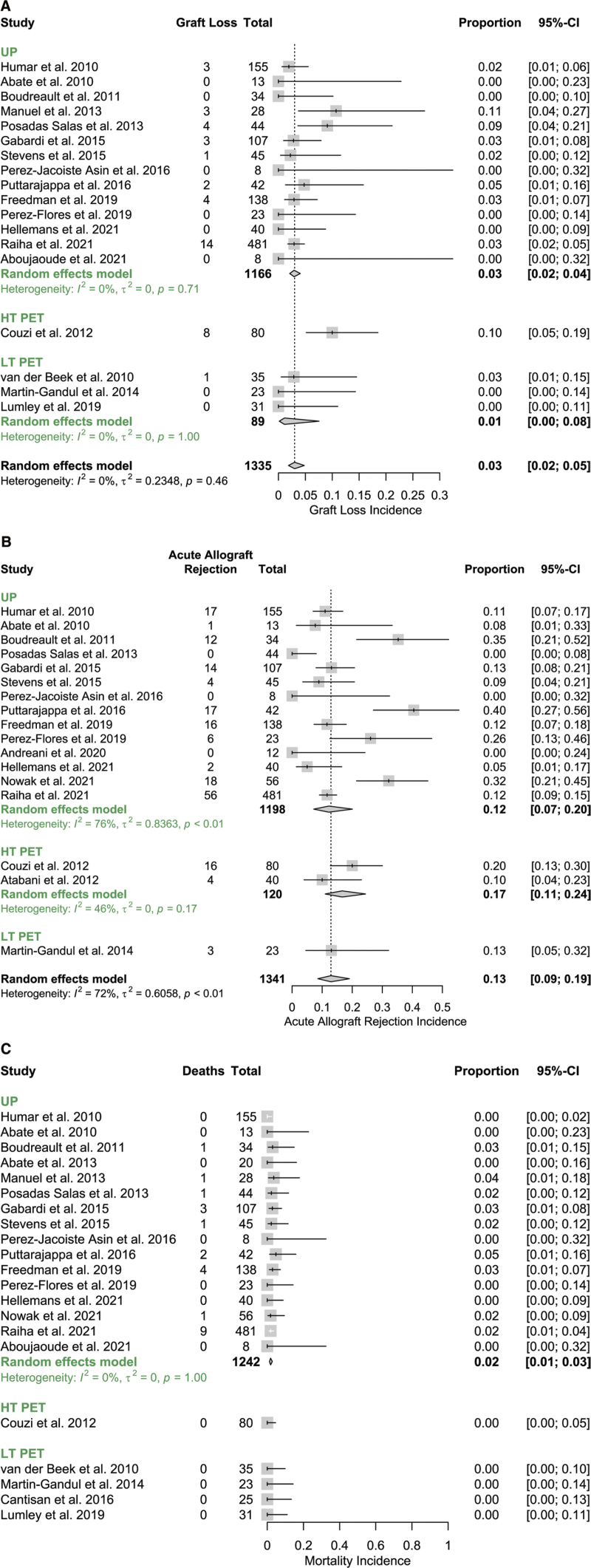
Forest plots showing the association of CMV preventive strategy with other clinical outcomes. A, Graft loss by 12-mo posttransplant. B, Acute allograft rejection (all types) by 12-mo posttransplant. C, Mortality by 12-mo posttransplant. CI, confidence interval; CMV, cytomegalovirus; HT, high threshold; LT, low threshold; PET, preemptive therapy; UP, universal prophylaxis.

There were no significant differences between pooled PET (HT and LT) and UP studies in any secondary outcome (*P* > 0.05 in all comparisons) (Figures 4A–C). Graft loss by 1-y was reported for UP in 14 of 19 studies (74%), for LT PET in 3 of 4 studies (75%), and for HT PET in 1 of 2 studies (50%) with an overall estimated incidence of 0.03 (95% CI, 0.02-0.05) (Figure 4A). A test for subgroup differences was significant (Q = 11.52, *p* = 0.003) and compared with UP, HT PET was significantly associated with higher incidence of graft loss $\left(\hat{\beta }=1.31,\;P=0.002\right)$ but was based on a single HT PET study. LT PET was not significantly associated with a difference in graft loss compared to UP $\left(\hat{\beta }=-0.97,\;P=0.34\right)$.

The incidence of acute allograft rejection was available for 14 of 19 (74%) UP studies and for 3 of 6 (50%) PET studies (1 LT-PET, 2 HT-PET), and the overall estimate of incidence was 0.13 (95% CI, 0.09-0.19) with moderate-high heterogeneity. A test of subgroup differences suggested no significant differences in acute allograft rejection incidence between the 3 prevention strategies (Q = 0.92, *p* = 0.6318), although there was a low number of PET studies included in this analysis (Figure 4B).

Mortality by 1-y was available for LT-PET in 4 of 4 studies (100%), for HT-PET in 1 of 2 studies (50%), and for UP in 16 of 19 studies (84%). Meta-analysis was not conducted in the PET studies for this outcome because there were no CMV events (0/194). The estimated 95% CI for mortality in PET studies is (0.00-0.02).^33^ Mortality in UP studies was 23 events in 1242 patients (incidence = 0.02 [95% CI, 0.01-0.03]) (Figure 4C).

### Publication Bias

**FIGURE 5.**
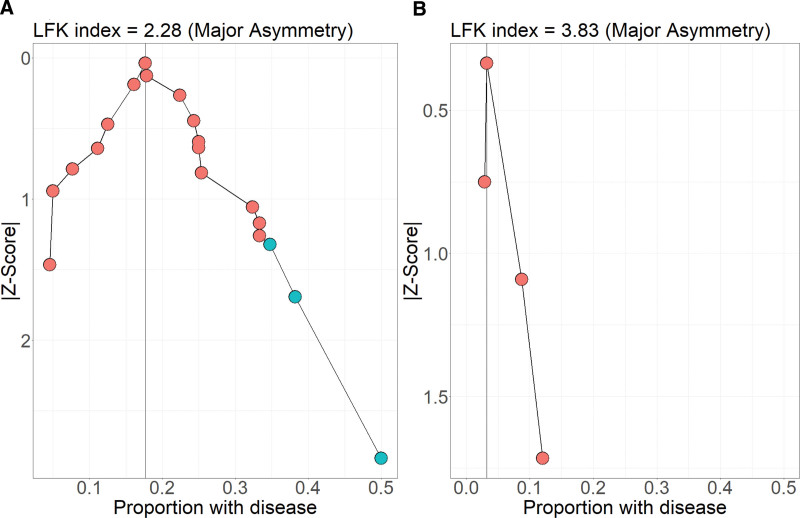
Doi plot assessing publication bias in (A) UP and (B) LT PET studies. Blue points represent the 3 studies that were removed to test robustness of results against publication bias. LT, low-threshold; PET, preemptive therapy; UP, universal prophylaxis.

Publication bias in UP studies was assessed using the Doi Plot and LFK index (Figure 5). An LFK index between −1 and 1 suggests low publication bias, whereas an LFK index outside this range suggests probable publication bias. The LFK indices were 2.28 and 3.83 for UP and LT PET respectively, suggesting a high probability of positive publication bias in both groups. This result indicates that the estimates of CMV disease incidence in both UP and LT PET may be skewed high due to publication bias. To assess whether the results were robust despite potential bias, we repeated the analysis after removing the 3 UP studies with the highest CMV disease incidence reported, yielding an LFK index of 0.622, which signifies a low probability of publication bias. Exclusion of these 3 studies resulted in a CMV disease incidence estimate of 0.19 (95% CI, 0.15-0.24) with lower, but still significant, heterogeneity (I^2^ = 42.7%, Q = 32.47, *P* = 0.006). Importantly, meta-regression with prevention strategy as a moderator variable still showed LT PET to be significantly associated with a lower CMV disease incidence compared to UP $\left(\hat{\beta }=-1.33,\;P=0.003\right)$.

## DISCUSSION

Our systematic review and meta-analysis of optimized PET and UP suggest that LT-PET (but not HT PET) is associated with a significantly lower incidence of CMV disease compared to UP with comparable incidence of other clinical outcomes in CMV D+R- KTRs. Conclusions from prior systematic reviews that PET and UP were comparable for prevention of CMV disease in CMV D+R- KTRs were likely affected by inclusion of both LT and HT PET studies within the PET group (and HT PET was associated with higher CMV disease incidence than LT PET). Our results provide preliminary data and compelling rationale to conduct a trial to assess the potential superiority of LT-PET over UP for prevention of CMV disease in CMV D+R- KTRs.

The primary outcome for the analyses, CMV disease (CMV syndrome or end-organ), is clinically important, associated with increased costs,^56-58^ linked with worse long-term patient and graft outcomes, and is the Food and Drug Administration-recommended primary outcome for CMV prevention studies in solid organ transplant recipients.^59^ An important and novel finding was that the efficacy of PET was significantly affected by the threshold used to initiate AVT in PET. The efficacy of PET was substantially higher with the use of a LTcompared with a HT for initiating AVT, which until now, has been speculated but not systematically assessed. This finding likely explains why prior meta-analyses that pooled LT and HT PET into a single PET group may have incorrectly concluded that PET and UP were comparable for CMV disease prevention. In studies of PET, the number of patients who received lymphocyte-depleting therapy was relatively small, but there was no significant difference in the incidence of CMV disease with or without LDA among those who received either PET or UP. This finding suggests that a rigorous prevention strategy may mitigate the increased risk for CMV conferred by LDA and is consistent with a post hoc analysis of the CMV Antiviral Prevention Strategies in D+R- Liver Transplants trial in which baseline receipt of antithymocyte globulin induction was not associated with an increased risk of CMV infection or disease in the PET group.^60^ Collectively, these findings suggest that PET may be effective in CMV D+R- KTRs who receive lymphocyte-depleting induction immunosuppression, but this should be confirmed in future clinical trials.

Observational and nonrandomized studies of interventions were generally at low-medium risk of bias for the primary outcome of CMV disease, which is to be expected for the study design. Four UP studies exhibited high/serious risk of bias, 2 of which were classified as such because of a low sample size. The other 2 studies were deemed to be at high risk of bias because of study designs in which patients were enrolled at any point within 1-y posttransplant, but only followed up to 1-y posttransplant. Thus, some CMV disease episodes within 1-y posttransplant may have been missed if they had occurred before enrollment. However, because this would bias toward the null (CMV disease incidence not different between UP and LT PET), there is less concern that this bias affects the primary finding that LT-PET was associated with a lower CMV disease incidence compared with UP.

In addition to CMV disease, randomized studies^61-64^ and meta-analyses have reported conflicting results regarding the association of CMV prevention strategy with other important clinical outcomes such as mortality or graft survival, with most studies reporting no association between CMV prevention strategy in clinical outcomes.^11,62,63^ In our analysis restricted only to optimized UP and PET studies, the rates of other clinical outcomes were generally similar between LT PET and UP. HT PET was associated with a higher rate of allograft loss compared with UP but this result was based on only a single HT PET study and requires further examination. CMV viremia has previously been shown to be a risk factor for graft loss in KTRs.^65^ Because the HT-PET group comprised a substantial component of the PET group in prior meta-analyses, it is possible that lower allograft survival with PET compared with UP was driven by inclusion of HT-PET studies in the PET group in those studies. Only well-powered randomized controlled trials of LT-PET and UP in CMV D+R- KTRs that include longer-term follow-up can clarify the relative effect of PET and UP on other important clinical outcomes.

An important and unexpected finding was that even among high-risk CMV D+R- KTRs, donor-transmitted CMV occurred only in ~65% of recipients, as assessed by frequent CMV DNAemia monitoring in the PET group. These results are nearly identical to those reported in a large single-center study that specifically sought to determine the rate of CMV transmission in CMV D+R- KTRs receiving UP.^66^ This suggests that CMV transmission occurs only in a proportion of, rather than universally among, D+R- KTRs.^66^ The implications of incomplete CMV transmission are that, with the use of PET in CMV D+R- KTRs, approximately one-third of patients would not have CMV transmission and would therefore be spared from the potential risks, costs, and toxicities of AVT compared with UP, in which all D+R- patients receive AVT.

Study strengths included a comprehensive, systematic review of existing literature, use of prespecified eligibility criteria for study inclusion, assessment of clinically relevant direct and indirect outcomes, and use of rigorous statistical methods for meta-analysis and interpretation. We acknowledge potential limitations. The definitions and details of CMV disease were not standardized and consistently reported across studies and might have differed from an endpoint adjudication committee.^67,68^ Although we identified substantial heterogeneity in the incidence of CMV disease in the prophylaxis studies, it was below the range reported in prior meta-analyses.^69^ Importantly, there was minimal heterogeneity in the LT-PET studies. Because DNAemia occurs more commonly with PET and is a key component in the diagnosis of CMV syndrome, there would be a potential bias for reporting a higher incidence of CMV disease (ie, DNAemia + variable symptoms) with PET rather than UP, but this was opposite to what we found. The rates of non-CMV disease clinical outcomes were low, limiting the ability to detect small differences, but this is consistent with the rates seen in the modern era of transplantation. The 1-y duration of follow-up may have been too short to detect differences over the longer term. However, in longer-term follow-up of 2 Randomized controlled trials comparing UP and PET, outcomes were either comparable or superior in the PET cohorts.^70,71^ The numbers who received LDA in the PET cohorts were small, so future studies to confirm these findings are needed. Cost analyses were not reported. Logistic considerations such as the need for frequent CMV monitoring and initiation of PET are potential limitations of PET and may not be applicable to all settings. However, reports of successful PET in a “real-world” setting provide reassurance for the feasibility of PET.^72^ Additionally, other important outcomes such as antiviral resistance and cost of care were reported in only a small number of studies, precluding definitive comparisons between UP and PET.

In summary, we found that LT-PET appeared to be more effective than HT PET and 6-mo VGCV UP for prevention of CMV disease in D+R- KTRs with comparable incidence of other clinical outcomes. These findings provide preliminary data and compelling rationale to conduct a randomized controlled trial to test the hypothesis that LT-PET is superior to UP for prevention of CMV disease in CMV D+R- KTRs.

## ACKNOWLEDGMENTS

The authors thank and acknowledge the following individuals for working to assist our project by providing us with additional data and/or clarifications from their published studies on CMV prevention: Dr Julie Ishida, Dr Roy Bloom, Dr Emily Blumberg, Dr Alden Doyle, Dr Martha van der Beek, Dr Julian Torre Cisneros, Dr Sara Cantisan, Dr Elisa Cordero, Dr Pierre Merville, Dr Lionel Couzi, Dr Hannah Kaminski, Dr Jennifer Trofe-Clark, Dr Tomas Reischig, Dr Joseph Kim, Dr Sasan Hosseini, Dr Jeffrey Yin, Dr Eric Claas, Dr James Mccaffrey, Dr Shenoy Mohan, Dr Chethan Puttarajappa, Dr Maroun Aboujaoude, Dr David Abate, Dr Isabel Perez-Flores, Dr Maria Posadas Salas, Dr Paul Griffiths, Dr Guiseppe Gerna, Dr Rachel Hellemans, Dr Ilkka Helantera, Dr Sowsan Atabani, Dr Juulia Grasberger, Dr Spenser January, Dr Johan Noble, Dr Oriol Manuel, Dr Mario Fernandez-Ruiz, Dr Hartmuth Nowak, Dr Megan Rech. They also thank Mrs Anna Liss Jacobsen, Mrs Electra Enslow, and Mrs Teresa Jewell for their assistance in crafting and refining our literature search strategies.

## Supplementary Material
