# Supplementary material for: A Systematic Review and Meta-analysis of Optimized CMV Preemptive Therapy and Antiviral Prophylaxis for CMV Disease Prevention in CMV High-Risk (D+R-) Kidney Transplant Recipients
Source: Transplant Direct. 2023 Jul 12;9(8):e1514. doi: 10.1097/TXD.0000000000001514 (PMC10348730; doi:10.1097/TXD.0000000000001514)
Supplement: Supplementary file 1 [file txd-9-e1514-s001.pdf]

Supplemental Digital Content

Table S1. Assay and modeled AVT initiation threshold for all PET studies.

PET category	Paper	Assay	Threshold for AVT initiation[a] (IU/mL)
HT PET			
	Couzi et al, 2012	TaqMan on LightCycler 1.0	1078[b]
	Atabani et al, 2012	In-house Taqman-based assay	1522
LT PET			
	van der Beek et al, 2010	In-house assay	100[c]
	Martin-Gandul et al, 2014	Quant CMV LightCycler 2.0 from October 2008 to April 2012 and Cobas Ampliprep/Cobas Taqman CMV test after	137
	Cantisan et al, 2016	COBAS AmpliPrep/COBAS TaqMan, Roche Diagnostics	137
	Lumley et al, 2019	Lab developed: Qiagen kit; Taqman Probe; ABI7700 thermal cycler	100

[a] Thresholds shown in IU/mL in plasma. Studies for which the threshold was reported in copies/mL and a conversion factor for copies/mL to IU/mL was not available were assumed to use the conversion factor 1 copy/mL = 1 IU/mL. Thresholds for whole blood were modeled in plasma using two methods and the average resulting plasma threshold was reported.

[b] Data from Mengelle et al, 2003.

[c] Data from Kalpoe et al, 2004.

AVT, antiviral therapy; CMV, cytomegalovirus; HT, high threshold; IU, international units; LT, low threshold; LOD, limit of detection; PET, preemptive therapy; PCR, polymerase chain reaction; WB, whole blood.

Table S2. Risk of bias in included randomized-controlled trials.

Study (N = 2)	Randomization process	Deviations from intended interventions	Missing outcome data	Measurement of the outcome	Selection of the reported result	Overall Risk of Bias assessment
Humar et al, 2010[a]	Low	Low	Low	Low	Low	Low
Fleming et al, 2017[a]	Low	Low	Low	Some concerns	Low	Some concerns

[a]Universal prophylaxis.

Table S3. Risk of bias in nonrandomized trials of interventions.

Study (N = 9)	Confounding	Selection of participants	Classification of interventions	Deviations from intended interventions	Missing data	Measurement of outcomes	Selection of the reported result	Overall
Leone et al, 2010[a]	Moderate	Low	Low	Low	Low	Moderate	Low	Moderate
Abate et al, 2010[a]	Moderate	Serious	Low	Low	Low	Low	Low	Serious
Manuel et al, 2013[a]	Low	Low	Low	Low	Low	Low	Low	Low
Gabardi et al, 2015[a]	Low	Low	Low	Low	Low	Moderate	Low	Moderate
Stevens et al, 2015[a]	Moderate	Low	Low	Low	Low	Low	Low	Moderate
van der Beek et al, 2010[b]	Moderate	Low	Low	Low	Moderate	Low	Low	Moderate
Cantisan et al, 2016[b]	Moderate	Low	Low	Low	Low	Moderate	Low	Moderate
Lumley et al, 2019[b]	Moderate	Low	Low	Low	Low	Low	Low	Moderate
Couzi et al, 2012[c]	Moderate	Low	Low	Low	Low	Moderate	Low	Moderate

[a]Universal prophylaxis.
[b]Low-threshold preemptive therapy.
[c]High-threshold preemptive therapy.

Table S4. Risk of bias in observational studies with no intervention.

Study (N = 13)	Q1	Q2	Q3	Q4	Q5	Q6	Q7	Q8	Q9	Q10	Overall risk
Bouldreault et al, 2011[a]	Yes	Yes	Yes	Yes	Yes	Yes	Yes	Yes	Yes	NA	Low
Abate et al, 2013[a]	Yes	No	No	Yes	Yes	Yes	Yes	Yes	Yes	Yes	High
Perez-Jacoiste Asin et al, 2016[a]	Yes	Yes	No	Yes	Yes	Yes	Yes	Yes	Yes	NA	High
Puttarajappa et al, 2016[a]	No	Yes	Yes	Yes	Yes	Unclear	Unclear	Yes	Yes	Yes	Moderate
Freedman et al, 2019[a]	No	Yes	Yes	Yes	Yes	Yes	Yes	Yes	Yes	Yes	Low
Perez-Flores et al, 2019[a]	No	Yes	No	Yes	Yes	Yes	Yes	Yes	Yes	Yes	Moderate
Andreani et al, 2020[a]	Yes	Yes	No	Yes	Yes	Yes	Yes	Yes	Yes	Yes	Moderate
Hellemans et al, 2021[a]	Yes	Yes	Yes	Yes	Yes	Yes	Yes	Yes	Yes	Yes	Low
Nowak et al, 2021[a]	Yes	Yes	Yes	Yes	Yes	Yes	Yes	Yes	Yes	Yes	Low
Raiha et al, 2021[a]	Yes	Yes	Yes	Yes	Yes	Yes	Yes	Yes	Yes	Yes	Low
Aboujaoude et al, 2021[a]	No	Yes	No	Yes	Yes	Unclear	Yes	Yes	Yes	Yes	High
Martin-Gandul et al, 2014[b]	Yes	Yes	No	Yes	Yes	Yes	Yes	Yes	Yes	Yes	Moderate
Atabani et al, 2012[c]	Yes	Yes	Yes	Yes	Yes	Yes	Yes	Yes	Yes	Yes	Low

[a]Universal prophylaxis.
[b]Low-threshold preemptive therapy.
[c]High-threshold preemptive therapy.

Figure S1. Search strategies used to query Pubmed and Embase databases.

Search strategy		Results
**Pubmed**		
1.	("Kidney Transplantation"[Mesh]) AND ("Cytomegalovirus"[Mesh] OR "Cytomegalovirus Infections"[Mesh]) AND ("Valganciclovir"[Mesh] OR "Ganciclovir"[Mesh]) NOT ("Child"[Mesh]) + Filters (Article types: Classical article, clinical Study, Clinical Trials, Introductory Journal Article, Multicenter Study, Observational Study, Species: Human, Language: English)	217
2.	((((("Epidemiologic Studies"[Mesh] OR "Case-Control Studies"[Mesh] OR "Cohort Studies"[Mesh] OR "cohort study"[tw] OR "cohort studies"[tw] OR "cohort analysis"[tw] OR "cohort analyses"[tw] OR "follow up study"[tw] OR "follow up studies"[tw] OR "observational study"[tw] OR "observational studies"[tw] OR longitudinal[tw] OR retrospective[tw] OR "cross sectional"[tw] OR "Cross-Sectional Studies"[Mesh])) AND ((((("kidney transplant"[Title]) OR "kidney transplantation"[Title]) AND "cytomegalovirus"[Title])) OR ((kidney transplantation[MeSH Terms]) AND cytomegalovirus[MeSH Terms]))) AND "last 10 years"[PDat])) OR (((("Controlled Clinical Trial"[All Fields] OR "Randomized Controlled Trial"[All Fields] OR "Clinical Trial"[All Fields] OR "Clinical Trial, Phase IV"[All Fields] OR "Clinical Trial, Phase III"[All Fields] OR "Clinical Trial, Phase II"[All Fields] OR "Clinical Trial, Phase I"[All Fields] OR "Multicenter Study"[All Fields] OR random*[tw] OR placebo[All Fields] OR trial[All Fields] OR groups[ti] OR "Clinical Trials as Topic"[Mesh] OR "Randomized Controlled Trials as Topic"[Mesh] OR "Double-Blind Method"[Mesh] OR "Single-Blind Method"[Mesh] OR ((singl*[tw] OR doubl*[tw] OR trebl*[tw] OR tripl*[tw]) AND (mask*[tw] OR blind*[tw] OR dumm*[tw])) OR "Random Allocation"[Mesh] OR random allocation[All Fields] OR open-label[All Fields] OR open-labeled[All Fields] OR open-labelled[All Fields] OR "Placebos"[Mesh] OR "Cross-Over Studies"[Mesh]))) AND ((((("kidney transplant"[Title]) OR "kidney transplantation"[Title]) AND "cytomegalovirus"[Title])) OR ((kidney transplantation[MeSH Terms]) AND cytomegalovirus[MeSH Terms])))	297
Final	[Search 1] OR [Search 2]	440
**Embase**		
1.	('kidney graft'/exp OR 'graft, kidney':ti,ab,kw OR 'kidney graft':ti,ab,kw OR 'kidney transplant':ti,ab,kw OR 'renal graft':ti,ab,kw OR 'renal transplant':ti,ab,kw OR 'transplant, kidney':ti,ab,kw OR 'kidney allograft'/exp OR 'allograft, kidney':ti,ab,kw OR 'homograft, kidney':ti,ab,kw OR 'kidney allograft':ti,ab,kw OR 'kidney homograft':ti,ab,kw OR 'kidney homotransplant':ti,ab,kw OR 'renal allograft':ti,ab,kw OR 'renal homograft':ti,ab,kw OR 'renal homotransplant':ti,ab,kw) AND	291

	('cytomegalovirus'/exp OR 'cytomegalovirus':ti,ab,kw OR 'cytomegaly virus':ti,ab,kw OR 'cmv virus':ti,ab,kw OR 'cytomegalia virus':ti,ab,kw OR 'cytomegalic inclusion body herpetovirus':ti,ab,kw OR 'cytomegalic inclusion body virus':ti,ab,kw OR 'cytomegalic inclusion disease virus':ti,ab,kw OR 'cytomegalic inclusion virus':ti,ab,kw OR 'cytomegalic virus':ti,ab,kw OR 'cytomegalo herpetovirus':ti,ab,kw OR 'cytomegaloviruses':ti,ab,kw OR 'cytomegalus virus':ti,ab,kw OR 'cytomegalusvirus':ti,ab,kw OR 'porcine cytomegalic inclusion disease virus':ti,ab,kw) AND ('prophylaxis'/de OR 'disease prevention':ti,ab,kw OR 'disease prophylaxis':ti,ab,kw OR 'prevention, disease':ti,ab,kw OR 'preventive medication':ti,ab,kw OR 'preventive therapy':ti,ab,kw OR 'preventive treatment':ti,ab,kw OR 'prophylactic institution':ti,ab,kw OR 'prophylactic management':ti,ab,kw OR 'prophylactic medication':ti,ab,kw OR 'prophylactic therapy':ti,ab,kw OR 'prophylactic treatment':ti,ab,kw OR 'prophylaxis':ti,ab,kw OR 'health protection':ti,ab,kw OR 'preemptive therapy'/exp OR 'pre-emptive prevention':ti,ab,kw OR 'pre-emptive preventive intervention':ti,ab,kw OR 'pre-emptive prophylaxis':ti,ab,kw OR 'pre-emptive therapy':ti,ab,kw OR 'pre-emptive treatment':ti,ab,kw OR 'preemptive prevention':ti,ab,kw OR 'preemptive prevention therapy':ti,ab,kw OR 'preemptive prophylaxis':ti,ab,kw OR 'preemptive prophylaxis therapy':ti,ab,kw OR 'preemptive therapy':ti,ab,kw OR 'preemptive treatment':ti,ab,kw OR 'pre-exposure prophylaxis'/exp OR 'pre-exposure prophylaxis':ti,ab,kw) AND ('valganciclovir'/exp OR 'valganciclovir' OR 'valcyte' OR 'valganciclovir hydrochloride' OR 'valgancyclovir' OR 'ganciclovir'/exp OR '2` nor 2` deoxyguanosine' OR '2` nor deoxyguanosine' OR '2` nordeoxyguanosine' OR '759u77' OR '9 (1, 3 dihydroxy 2 propoxymethyl) guanine' OR '9 [(1, 3 dihydroxy 2 propoxy) methyl] guanine' OR '9 [[2 hydroxy 1 (hydroxymethyl) ethoxy] methyl] guanine' OR '9 [2 hydroxy 1 (hydroxymethyl) ethoxymethyl] guanine' OR 'b 759 u' OR 'b 759u' OR 'b759u' OR 'biolf 62' OR 'biolf62' OR 'bw 759' OR 'bw 759 u' OR 'bw 759u' OR 'bw 759u77' OR 'bw b759u' OR 'bw759' OR 'bw759u' OR 'bw759u77' OR 'bwb 759 u' OR 'bwb 759u' OR 'bwb759u' OR 'cameven' OR 'citovirax' OR 'cymevan' OR 'cymeven' OR 'cymevene' OR 'cytovene' OR 'cytovene iv' OR 'denocin' OR 'denosine' OR 'dihydroxypropoxymethylguanine' OR 'ganciclovir' OR 'ganciclovir sodium' OR 'gancyclovir' OR 'guanine, 9 [(1, 3 dihydroxy 2 propoxy) methyl]' OR 'rs 21592' OR 'rs21592' OR 'virgan' OR 'vitrasert' OR 'vitrasert implant' OR 'zirgan') AND ('high-risk' OR 'high risk' OR 'd+/r' OR 'seropositive' OR 'seronegative') AND [01-01-2000]/sd NOT [09-01-2022]/sd AND [2000-2022]/py	
2.	('kidney transplantation'/exp OR 'kidney transplant*':ab,ti) AND ('cytomegalovirus':ab,ti OR 'cmv':ab,ti) AND [english]/lim AND [humans]/lim AND ('clinical trial'/de OR 'controlled clinical trial'/de OR 'controlled study'/de OR 'randomized controlled trial'/de OR 'randomized controlled trial (topic)'/de OR 'cohort analysis'/de OR 'medical record review'/de OR 'observational study'/de OR 'prospective study'/de OR 'retrospective study'/de) NOT ('clinical protocol'/de OR 'human cell'/de OR 'human	194

	tissue'/de OR 'intermethod comparison'/de OR 'nonhuman'/de OR 'systematic review'/de) NOT ('conference abstract'/it OR 'conference paper'/it OR 'editorial'/it OR 'letter'/it OR 'note'/it OR 'review'/it OR 'short survey'/it) NOT ('pediatric' OR 'adolescent') AND ('valganciclovir' OR 'ganciclovir' OR 'vgcv' OR 'gcv') AND [01-01-2000]/sd NOT [09-01-2022]/sd AND [2000-2022]/py	
Final	#1 OR #2	437

Figure S2. PRISMA 2020 checklist.

PRISMA 2020 checklist

Section and Topic	Item #	Checklist item	Location where item is reported
**TITLE**			
Title	1	Identify the report as a systematic review.	1
**ABSTRACT**			
Abstract	2	See the PRISMA 2020 for Abstracts checklist.	4 - 5
**INTRODUCTION**			
Rationale	3	Describe the rationale for the review in the context of existing knowledge.	6 – 7
Objectives	4	Provide an explicit statement of the objective(s) or question(s) the review addresses.	6 – 7
**METHODS**			
Eligibility criteria	5	Specify the inclusion and exclusion criteria for the review and how studies were grouped for the syntheses.	7 - 8
Information sources	6	Specify all databases, registers, websites, organisations, reference lists and other sources searched or consulted to identify studies. Specify the date when each source was last searched or consulted.	7
Search strategy	7	Present the full search strategies for all databases, registers and websites, including any filters and limits used.	S Fig 1
Selection process	8	Specify the methods used to decide whether a study met the inclusion criteria of the review, including how many reviewers screened each record and each report retrieved, whether they worked independently, and if applicable, details of automation tools used in the process.	8
Data collection process	9	Specify the methods used to collect data from reports, including how many reviewers collected data from each report, whether they worked independently, any processes for obtaining or confirming data from study investigators, and if applicable, details of automation tools used in the process.	9
Data items	10a	List and define all outcomes for which data were sought. Specify whether all results that were compatible with each outcome domain in each study were sought (eg, for all measures, time points, analyses), and if not, the methods used to decide which results to collect.	9 - 10
	10b	List and define all other variables for which data were sought (eg, participant and intervention characteristics, funding sources). Describe any assumptions made about any missing or unclear information.	9 - 10
Study risk of bias assessment	11	Specify the methods used to assess risk of bias in the included studies, including details of the tool(s) used, how many reviewers assessed each study and whether they worked independently, and if applicable, details of automation tools used in the process.	10
Effect measures	12	Specify for each outcome the effect measure(s) (eg, risk ratio, mean difference) used in the synthesis or presentation of results.	10
Synthesis methods	13a	Describe the processes used to decide which studies were eligible for each synthesis (eg, tabulating the study intervention characteristics and comparing against the planned groups for each synthesis (item #5)).	9
	13b	Describe any methods required to prepare the data for presentation or synthesis, such as handling of missing summary statistics, or data conversions.	10 - 11
	13c	Describe any methods used to tabulate or visually display results of individual studies and syntheses.	10

	13d	Describe any methods used to synthesize results and provide a rationale for the choice(s). If meta-analysis was performed, describe the model(s), method(s) to identify the presence and extent of statistical heterogeneity, and software package(s) used.	10 - 11
	13e	Describe any methods used to explore possible causes of heterogeneity among study results (eg, subgroup analysis, meta-regression).	10 - 11
	13f	Describe any sensitivity analyses conducted to assess robustness of the synthesized results.	11
Reporting bias assessment	14	Describe any methods used to assess risk of bias due to missing results in a synthesis (arising from reporting biases).	10 (included in instruments)
Certainty assessment	15	Describe any methods used to assess certainty (or confidence) in the body of evidence for an outcome.	10-11

Section and Topic	Item #	Checklist item	Location where item is reported
**RESULTS**			
Study selection	16a	Describe the results of the search and selection process, from the number of records identified in the search to the number of studies included in the review, ideally using a flow diagram.	Fig. 1
	16b	Cite studies that might appear to meet the inclusion criteria, but which were excluded, and explain why they were excluded.	12
Study characteristics	17	Cite each included study and present its characteristics.	Table 1
Risk of bias in studies	18	Present assessments of risk of bias for each included study.	Supp Table 2 - 4
Results of individual studies	19	For all outcomes, present, for each study: (a) summary statistics for each group (where appropriate) and (b) an effect estimate and its precision (eg, confidence/credible interval), ideally using structured tables or plots.	Fig 2 - 4
Results of syntheses	20a	For each synthesis, briefly summarise the characteristics and risk of bias among contributing studies.	Table 1, Supp table 2 – 4
	20b	Present results of all statistical syntheses conducted. If meta-analysis was done, present for each the summary estimate and its precision (eg, confidence/credible interval) and measures of statistical heterogeneity. If comparing groups, describe the direction of the effect.	12 – 14
	20c	Present results of all investigations of possible causes of heterogeneity among study results.	12 – 14
	20d	Present results of all sensitivity analyses conducted to assess the robustness of the synthesized results.	14 – 15
Reporting biases	21	Present assessments of risk of bias due to missing results (arising from reporting biases) for each synthesis assessed.	11
Certainty of evidence	22	Present assessments of certainty (or confidence) in the body of evidence for each outcome assessed.	Supp Table 2 - 4, 12 - 13
**DISCUSSION**			
Discussion	23a	Provide a general interpretation of the results in the context of other evidence.	15 – 16
	23b	Discuss any limitations of the evidence included in the review.	16 – 18

	23c	Discuss any limitations of the review processes used.	17 – 18
	23d	Discuss implications of the results for practice, policy, and future research.	15 – 18
**OTHER INFORMATION**			
Registration and protocol	24a	Provide registration information for the review, including register name and registration number, or state that the review was not registered.	9
	24b	Indicate where the review protocol can be accessed, or state that a protocol was not prepared.	9
	24c	Describe and explain any amendments to information provided at registration or in the protocol.	NA
Support	25	Describe sources of financial or nonfinancial support for the review, and the role of the funders or sponsors in the review.	2
Competing interests	26	Declare any competing interests of review authors.	2
Availability of data, code and other materials	27	Report which of the following are publicly available and where they can be found: template data collection forms; data extracted from included studies; data used for all analyses; analytic code; any other materials used in the review.	9

From: Page MJ, McKenzie JE, Bossuyt PM, et al. The PRISMA 2020 statement: an updated guideline for reporting systematic reviews. *BMJ*. 2021;372:n71.

For more information, visit: http://www.prisma-statement.org/
